# The Role of *EjSOC1*s in Flower Initiation in *Eriobotrya japonica*

**DOI:** 10.3389/fpls.2019.00253

**Published:** 2019-03-04

**Authors:** Yuanyuan Jiang, Jiangrong Peng, Yunmei Zhu, Wenbing Su, Ling Zhang, Yi Jing, Shunquan Lin, Yongshun Gao

**Affiliations:** ^1^State Key Laboratory for Conservation and Utilization of Subtropical Agro-Bioresources, College of Horticulture, South China Agricultural University, Guangzhou, China; ^2^BGI Genomics, BGI-Shenzhen, Shenzhen, China

**Keywords:** loquat, flowering time, GA_3_, short-day, *EjSOC1*, *EjAP1*, *EjLFY*

## Abstract

The MADS-box transcription factor SUPPRESSOR OF OVEREXPRESSION OF CONSTANS1 (*SOC1*) integrates environmental and endogenous signals to promote flowering in *Arabidopsis*. However, the role of *SOC1* homologs in regulating flowering time in fruit trees remains unclear. To better understand the molecular mechanism of flowering regulation in loquat (*Eriobotrya japonica* Lindl.), two *SOC1* homologs (*EjSOC1-1* and *EjSOC1-2*) were identified and characterized in this work. Sequence analysis showed that EjSOC1-1 and EjSOC1-2 have conserved MADS-box and K-box domains. *EjSOC1-1* and *EjSOC1-2* were clearly expressed in vegetative organs, and high expression was detected in flower buds. As observed in paraffin-embedded sections, expression of the downstream flowering genes *EjAP1*s and *EjLFY*s started to increase at the end of June, a time when flower bud differentiation occurs. Additionally, high expression of *EjSOC1-1* and *EjSOC1-2* began 10 days earlier than that of *EjAP1*s and *EjLFY*s in shoot apical meristem (SAM). *EjSOC1-1* and *EjSOC1-2* were inhibited by short-day (SD) conditions and exogenous GA_3_, and flower bud differentiation did not occur after these treatments. EjSOC1-1 and EjSOC1-2 were found to be localized to the nucleus. Moreover, ectopic overexpression of *EjSOC1-1* and *EjSOC1-2* in wild-type *Arabidopsis* promoted early flowering, and overexpression of both was able to rescue the late flowering phenotype of the *soc1-2* mutant. In conclusion, the results suggest that cultivated loquat flower bud differentiation in southern China begins in late June to early July and that *EjSOC1-1* and *EjSOC1-2* participate in the induction of flower initiation. These findings provide new insight into the artificial regulation of flowering time in fruit trees.

## Introduction

Plant evolution has resulted in a variety of endogenous and exogenous factors that form a complex and sophisticated regulatory network to accurately respond to internal and external signals and integrate them to promote blooming at the most favorable time. The molecular genetic mechanisms at play in annual flowering plants, such as *Arabidopsis thaliana*, involve multiple regulatory pathways, including photoperiod, age, autonomic, vernalization, and gibberellin pathways (Moon et al., 2005; Amasino, 2010; Srikanth and Schmid, 2011; Teotia and Tang, 2015). These pathways precisely regulate flowering in *Arabidopsis* through major integrated genes such as *FLOWERING LOCUS T (FT)*, *LEAFY (LFY)*, and *SUPPRESSOR OF OVEREXPRESSION OF CONSTANS1 (SOC1)*.

MADS-box genes are a key components of flower development networks. In addition to the MADS-box domain, MIKC^C^-type MADS-box genes contain three other domains, the I-domain, K-box and C-terminal domain; although the MADS-box is highly conserved, the degree of conservation of the I-domain and C-domain is relatively low (Theissen et al., 1996; Parenicova et al., 2003; Vandenbussche et al., 2003; Smaczniak et al., 2012; Chen et al., 2017). *SOC1* is a member of the MIKC^C^-type gene family and encodes a type II MADS-box protein that contains the highly conserved MADS-box, K-box, and a C-terminal SOC1 motif (Vandenbussche et al., 2003). *SOC1* plays a vital role in regulating plant development and flower organogenesis by integrating photoperiod, age, and gibberellin signals (Parcy, 2005; Lee and Lee, 2010; Teotia and Tang, 2015).

*SOC1* is also found in other plants, such *Oryza sativa* (Tadege et al., 2003), *Petunia hybrida* (Ferrario et al., 2004), *Citrus sinensis* (Tan and Swain, 2007), *Glycine max* (Zhong et al., 2012), *Fragaria vesca* (Mouhu et al., 2013), *Zea mays* (Zhao et al., 2014), *Brassica juncea* (Sri et al., 2015), *Actinidia* spp. (Voogd et al., 2015), *Kalanchoe daigremontiana* (Liu et al., 2016), and *Mangifera indica* L. (Wei et al., 2016). *SOC1* not only promotes flowering but also regulates other biological functions, such as floral organ identity deterioration in *Gerbera hybrid* (Ruokolainen et al., 2011), repression of flowering and promotion of vegetative growth in *F. vesca* (Mouhu et al., 2013), and dormancy duration in kiwifruit (Voogd et al., 2015). *SOC1* function can vary among different plant species, though the function of *EjSOC1* in loquat has not been studied.

Loquat (*Eriobotrya japonica* Lindl.) is an evergreen fruit tree belonging to the family Rosaceae that is cultivated mainly in tropical and subtropical regions. In Rosaceae, flower initiation and flowering typically occur in different years in species including apple, pear, plum, strawberry, and raspberry (Kurokura et al., 2013). However, flower bud initiation and flowering occur within the same year in loquat, with the former generally occurring from July to September in China (Lin, 2007) and the latter mainly from October to January; there is also slight variability depending on the cultivar and environment. To date, 26 *Eriobotrya* species have been identified, and each wild species has a different flowering time that includes the months of November to June of the next year for some (Lin, 2017). For example, cultivated loquat (*E. japonica* Lindl.) blooms in fall or early winter, whereas *E. deflexa* Nakai blooms from May to June (Gu and Spongberg, 2003).

Although the flowering of loquat has the above characteristics, there have been few reports on it. To date, several flower-related genes, such as *EjAP1 (APETALA1)*, *EjFT*, *EjLFY*, and *EjTFL1* (*TERMINAL FLOWER1*) (Esumi et al., 2005; Liu et al., 2013, 2017; Reig et al., 2017), have been cloned from cultivated loquat, with *EdFT* and *EdFD (FLOWERING LOCUS D)* cloned from wild loquat (*E. deflexa* Nakai forma *koshunensis*) (Zhang L. et al., 2016).

In this study, the flower initiation time of cultivated loquat (“Jiefangzhong”) in Southern China was confirmed. Two *SOC1*-like genes from cultivated loquat were identified and named *EjSOC1-1* and *EjSOC1-2*. To elucidate their roles in regulating flowering time in loquat, their expression patterns and subcellular localizations were analyzed. In addition, we examined their function using transgenic *Arabidopsis* and explored the effects of short-day (SD) and GA_3_ treatments on bud differentiation.

## Materials and Methods

### Plant Materials and Growth Conditions

Material was collected from 12-year-old “Jiefangzhong” loquat (*E. japonica* Lindl.) trees grown under natural conditions in the loquat germplasm resource preservation garden, South China Agricultural University, Guangzhou, China (N23°09′N,113°20′E). The trees used in the experiments were grafted, and they had grown to the flowering stage. Leaf and shoot apical meristem (SAM) tissues were randomly sampled from three sites on the trees, and tissues was collected at 16:00. Wild-type *A. thaliana* ecotype Col-0 and the *soc1-2* mutant were used for genetic transformation. *Nicotiana benthamiana* was grown for transient expression. *Arabidopsis* and *Nicotiana* were grown under long-day conditions (16 h light/8 h dark) at 22°C.

### RNA Isolation, cDNA Preparation, Gene Isolation, and Sequence Analysis

Frozen mature loquat leaves or other tissues were ground to a powder in a mortar with liquid nitrogen. Total RNA was extracted using EasySpin Plus (Aidlab, China) and digested with recombinant RNase-free DNase I (Aidlab, China). First-strand cDNA was generated from loquat leaf RNA using the PrimeScript^TM^ RT (TAKARA, Japan) reagent kit and gDNA Eraser (TAKARA, Japan), the experiment was proceeded according to the manufacturer’s instructions.

The full-length coding sequences of *EjSOC1-1* and *EjSOC1-2* were obtained from the completed loquat *de novo* genome sequencing project, which has not yet been published. The two sequences were isolated from mature loquat leaf cDNA using Phusion DNA Polymerase (TAKARA, Japan). The gene-specific primers used for cloning were listed in Supplementary Table S1. Alignment of the deduced protein sequences was performed using ClustalX 2.0.12 and GeneDoc 2.7. Phylogenetic trees were constructed with MEGA 6.06 using the Neighbor-Joining (N-J) method with 1,000 bootstrap replicates.

### Gene Expression Analysis

Primers for qPCR were designed using Primer 5 software, and their specificity was confirmed by melting curve analysis and sequencing. qPCR was carried out in triplicate using a LightCycler^®^ 480 system (Roche, United States) with iTaq^TM^ universal SYBR Green Supermix (Bio-Rad, United States). The relative expression levels of target genes were evaluated using the ΔΔCt (cycle threshold) method. *β*-Actin was used as an internal reference gene for loquat (Shan et al., 2008). *AtPP2AA3* (AT1G13320) was used as an internal control for *Arabidopsis* (Hong et al., 2010). Semi-quantitative reverse transcription PCR (RT-PCR) was used for detecting exogenous gene expression in transgenic *Arabidopsis* lines. The primers used for RT-PCR were identical to the cloning primers (removal of the stop codon). The primers used are listed in Supplementary Table S2.

### Vector Construction

For construction of *35S:EjSOC1-1/EjSOC1-2-6HA* and *35S:EjSOC1-1/EjSOC1-2-GFP* plasmids, coding regions without the stop codon were cloned into pGreen-35S-6HA (Hou et al., 2014) and pGreen-35S-GFP (Lee et al., 2012), respectively. All primers used for vector construction are listed in Supplementary Table S3. The constructed plasmids were verified by sequencing and introduced into *Agrobacterium tumefaciens* strain *GV3101::psoup*.

### *Arabidopsis* Transformation

*35S:EjSOC1-1-HA* and *EjSOC1-2-HA* were introduced into *Agrobacterium tumefaciens GV3101::psoup* and then transformed into *Arabidopsis* Col-0 using the floral dip method (Zhang et al., 2006). Transgenic lines were screened on soil by Basta. For each construct, more than 10 independent transgenic lines were screened out, and two homozygous T3 generation lines of each genotype were used for checking ectopic gene expression.

### Short-Day and GA_3_ Treatments

An awning (Supplementary Figure S1) was set up to cover the tree to provide 8 h (10:00–18:00) of natural light and 16 h of darkness (18:00–10:00 [the next day]) each day. Control trees were grown under normal conditions. The experimental period lasted from May 18th to August 10th.

For GA_3_ treatment, trees were sprayed with 300 mg L^-1^ GA_3_ (Guangzhou DingGuo Biology Company, China) aqueous solution containing 0.1% (v/v) phosphoric acid and 0.025% (v/v) Triton X-100 as a surfactant. Control trees were sprayed with a solution containing only 0.1% (v/v) phosphoric acid and 0.025% (v/v) Triton X-100. All leaves and top buds were sprayed every 2 weeks from May 18th to August 10th.

### Subcellular Localization Analysis

*Agrobacterium*-mediated transient transformation of *N. benthamiana* leaves (Sparkes et al., 2006) was used to observe the subcellular localization of EjSOC1-1 and EjSOC1-2. Green fluorescent protein (GFP) fluorescence signals were detected using a fluorescence microscope Observer D1 (Zeiss, Germany). A GFP-free construct was used as a negative control.

### Data Analysis

Significant differences between data were evaluated by Student’s *t*-test. Calculations were carried out using GraphPad Prism 6 software.

## Results

### Observation of Flowering and Determination of Flowering Initiation in Loquat

**FIGURE 1:**
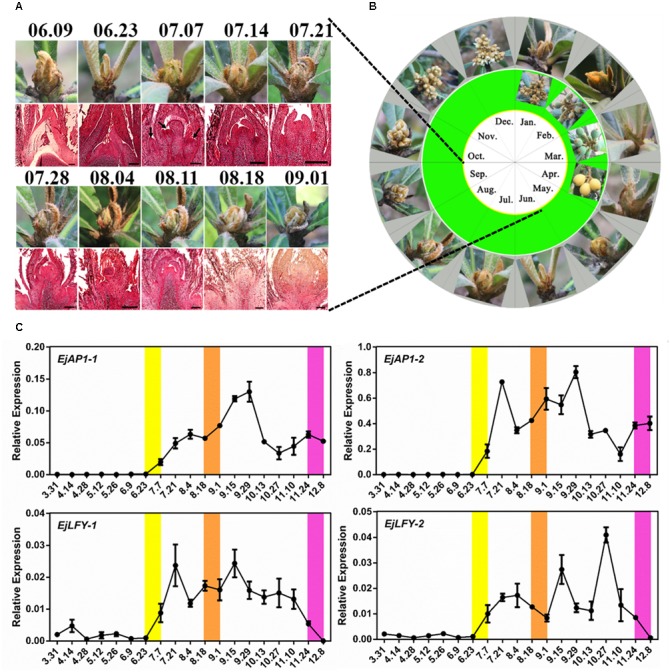
Determination of the flowering time of “Jiefangzhong” loquat. **(A)** The morphology of the shoot apex and its paraffin sections from June to September, with a scale of 200 μm. **(B)** The morphology of the shoot apex of loquat throughout the year. **(C)** Relative expression levels of the floral meristem identity genes *EjAP1*s and *EjLFY*s in the shoot apex (error bars indicating SD from three biological replicates). The yellow background represents the critical period of flower bud differentiation, and the orange background represents the period in which obvious inflorescence can be seen; the purple background represents the period of flower opening.

Continuous year-round observation of loquat SAM development was conducted. The panicle of “Jiefangzhong” loquat in Guangzhou was clearly visible from the end of August to early September (Figure 1A,B). In addition, analysis of paraffin-embedded sections of the SAM from June to September revealed no obvious inflorescence primordium formation in apical tissue before June 23rd, with multiple inflorescence primordia in the bottom of panicle appearing on July 7th (Figure 1A).

Furthermore, the expression levels of the floral meristem identity genes *EjAP1-1*, *EjAP1-2*, *EjLFY-1*, and *EjLFY-2* at different developmental stages of apical tissues were analyzed by qPCR. The results showed a high level of expression for both *EjAP1*s and *EjLFY*s began on June 23rd that was maintained from July to September (Figure 1C), except for *EjLFY-2*, which maintained a peak until November. *AP1* and *LFY* determine flower meristem characteristics and are key genes for flower induction and morphology (Lohmann et al., 2001). These results indicate that “Jiefangzhong” loquat flower bud differentiation in Guangzhou begins in late June to early July.

### Cloning and Identification of *SOC1*-Homologous Genes

**FIGURE 2:**
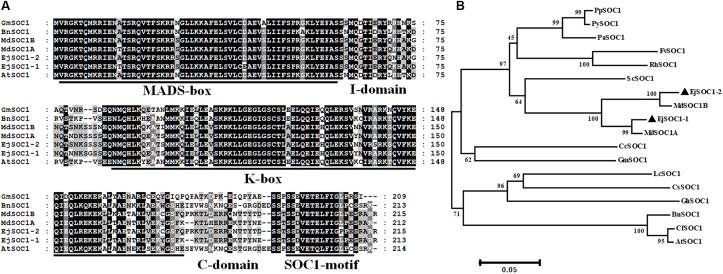
Sequence and phylogenetic analyses of EjSOC1-1 and EjSOC1-2. **(A)** Amino acid sequence alignment of several plant SOC1 family proteins. The highly conserved MADS-box, K-box, and SOC1-motif domains are marked by a solid line. The I-domain is located behind the MADS-box, and the C-domain is located behind the K-box. **(B)** Phylogenetic analysis of SOC1 family proteins from different species. The protein sequences of SOC1 genes aligned in this study were retrieved from NCBI. Accession IDs: AtSOC1 (NP_182090.1), BnSOC1 (NP_001303107.1), CcSOC1 (AHI85950.1), CsSOC1 (NP_001275772.1), CfSOC1 (AGN29205.1), FvSOC1 (AEO20231.1), GhSOC1 (AEA29618.1), GmSOC1 (NP_001236377.1), LcSOC1 (AGS32267.1), MdSOC1A (BAI49494.1), MdSOC1B (BAI49495.1), PaSOC1 (ACO40488.1), PpSOC1 (AJW29024.1), PySOC1 (AEO20233.1), and RhSOC1 (AEO20230.1).

We cloned two genes homologous to *SOC1*, *EjSOC1-1* and *EjSOC1-2*, using unpublished loquat genome sequence data. *EjSOC1-1* and *EjSOC1-2* CDSs are 642 and 648 bp and encode 213 and 215 amino acids, respectively (Supplementary Figure S2). Their sequences are highly similar, with nucleotide sequence identity of 93.36%. The predicted protein amino acid sequences of EjSOC1-1 and EjSOC1-2 are similar to those of other SOC1 orthologs from apple, soybean, rapeseed and *Arabidopsis* (Figure 2A). Sequence analysis showed that EjSOC1-1 and EjSOC1-2 harbor highly conserved MADS-box, K-box and SOC1-motif domains (Figure 2A).

Based on phylogenetic analysis of EjSOC1s and other plant SOC1 sequences, EjSOC1s and the other SOC1s from Rosaceae grouped into a large clade, with apple sequences forming a small clade with a high genetic relationship to the large clade (Figure 2B). EjSOC1s show the highest sequence similarity to MdSOC1 homologs (97.18% identity for EjSOC1-1 and MdSOC1A and 97.21% identity for EjSOC1-2 and MdSOC1B) (Figure 2A). These results confirm that EjSOC1-1 and EjSOC1-2 are MADS-box genes and SOC1 orthologs in loquat.

### Expression Analysis of *EjSOC1*s in Different Tissues

**FIGURE 3:**
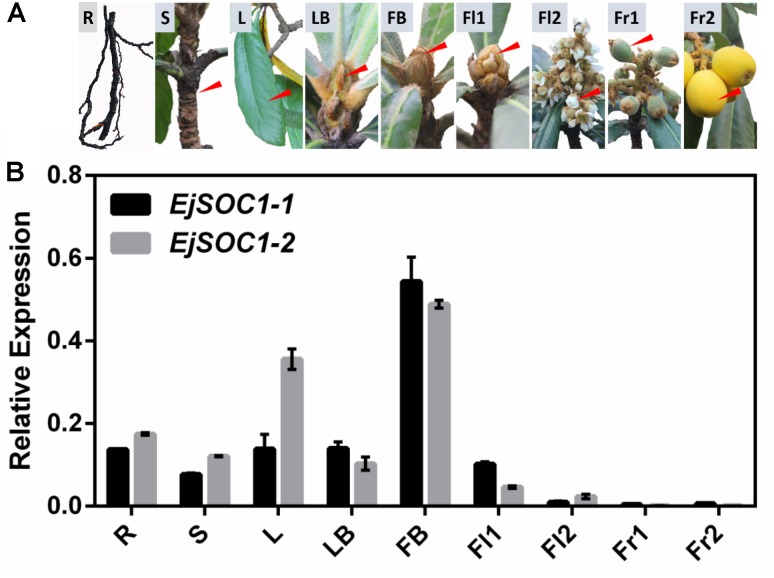
Tissue-specific expression of *EjSOC1-1* and *EjSOC1-2*. **(A)** Different tissues from loquat (*Eriobotrya japonica* Lindl.) were analyzed. **(B)** Relative expression of *EjSOC1*s in different tissues shown in panel **(A)** (error bars indicating SD from three biological replicates). The *β*-actin gene served as an internal control. R, root (from rootstock); L, leaf; S, shoot; LB, leaf buds (April 28th); FB, flower bud (July 21st); Fl1, flower 1 (September 29th); Fl2, flower 2 (December 8th); Fr1, fruit 1 (February 2nd) and Fr2, fruit 2 (April 13th).

To understand the potential function of *EjSOC1-1* and *EjSOC1-2* in loquat, we employed qPCR to examine the expression patterns of *EjSOC1-1* and *EjSOC1-2* in various tissues of “Jiefangzhong” loquat, including roots (from rootstock), leaves, shoots, leaf buds, flower buds, flowers, and fruits (Figure 3A). *EjSOC1-1* and *EjSOC1-2* were mainly expressed in vegetative organs, and for both, the highest expression was observed in flower buds. In particular, the expression level of *EjSOC1*s in early flower buds was significantly higher than that in blooming flowers, with scant expression in fruits (Figure 3B). These results suggest that *EjSOC1*s participate in the development of vegetative organs and flower initiation.

### Expression of EjSOC1s During the Growth and Development of Loquat

To further investigate the functions of *EjSOC1-1* and *EjSOC1-2* during vegetative and reproductive developmental processes, we examined their expression at different developmental stages of leaves, buds and flowers, as well as leaves of different maturities in the same period (Supplementary Figure S3), using qPCR.

**FIGURE 4:**
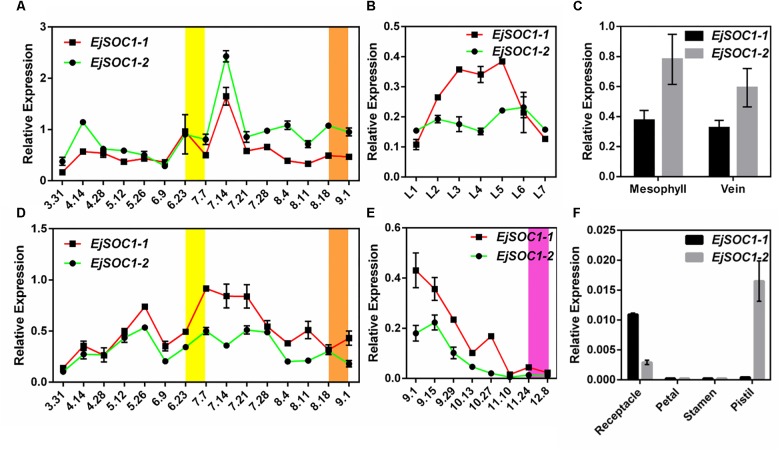
Relative expression patterns of *EjSOC1-1* and *EjSOC1-2*. **(A)** Relative expression patterns of *EjSOC1-1* and *EjSOC1-2* in different developmental stages of leaves. **(B)** Relative expression patterns of *EjSOC1-1* and *EjSOC1-2* in leaves at different levels of maturity in the same period. **(C)** Relative expression patterns of *EjSOC1-1* and *EjSOC1-2* in mesophyll tissue and veins. **(D)** Relative expression patterns of *EjSOC1-1* and *EjSOC1-2* in the SAM in different developmental periods. **(E)** Relative expression patterns of *EjSOC1-1* and *EjSOC1-2* in inflorescences at different developmental stages. **(F)** Relative expression patterns of *EjSOC1-1* and *EjSOC1-2* in different flower parts. Error bars indicating SD from three biological replicates.

In leaves, the tendency of *EjSOC1-1* expression was similar to that of *EjSOC1-2*: their expression began to increase on June 23rd and reached the highest level by July 14th (Figure 4A). In addition, there was no significant difference in expression between the genes in mesophyll tissue or in veins (Figure 4C).

With regard to the SAM in different periods, the expression levels of *EjSOC1-1* and *EjSOC1-2* began to increase sharply on June 23rd and reached the highest level around July 7th (Figure 4D). Moreover, expression of these genes gradually decreased as flower bud development progressed (Figure 4E).

For different flower parts (Supplementary Figure S4), *EjSOC1-1* and *EjSOC1-2* showed relatively high expression levels in receptacles, but only *EjSOC1-2* was highly expressed in pistils. Little expression of either was found in petals and stamens (Figure 4F).

In summary, *EjSOC1-1* and *EjSOC1-2* may function to induce flowering and are involved in the growth and development of early flower organs in loquat.

Interestingly, we found that *EjSOC1-1* and *EjSOC1-2* exhibited different expression trends in leaves at different levels of maturity in the same period. *EjSOC1-1* showed high expression in L3, L4 and L5 but relatively low expression in L1, L6, and L7 (Figure 4B), though *EjSOC1-2* did not display this trend. The results indicate that *EjSOC1-1* might also be involved in leaf development.

### *EjSOC1*s Are Inhibited by Short-Day and Exogenous GA_3_ Treatments

**FIGURE 5:**
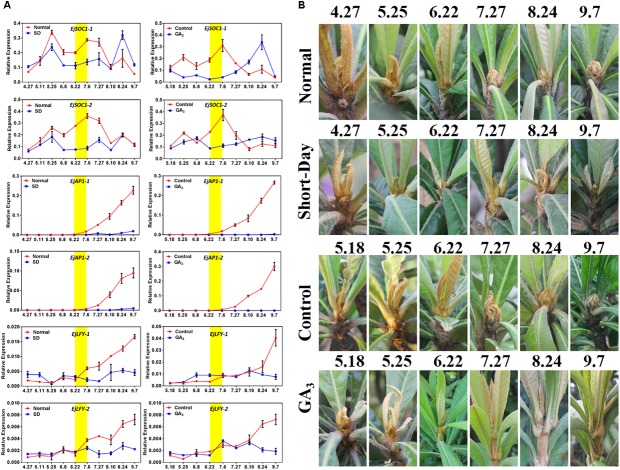
Effects of short-day and exogenous GA_3_ treatments on flowering. **(A)** Relative expression levels of *EjSOC1*s, *EjAP1*s, and *EjLFY*s in the shoot apex after different treatments (error bars indicating SD from three biological replicates). **(B)** The morphology of the shoot apex of loquat after different treatments.

After we analyzed the possible roles of *EjSOC1-1* and *EjSOC1-2* in loquat flowering, further exploration of the function of *EjSOC1*s under SD and exogenous GA_3_ was proceeded. *SOC1* can integrate the photoperiod and gibberellin pathways to regulate flower bud differentiation in *Arabidopsis*, and the loquat flower bud differentiation time coincides with the longest day of the year (summer solstice). Thus, we designed two experiments to alter growth conditions to explore whether *EjSOC1*s are affected by photoperiod and gibberellin. Interestingly, *EjSOC1-1* and *EjSOC1-2* were affected by SD and GA_3_ treatments, with lower levels of expression during the critical period of flower bud differentiation (late June and early July). *EjSOC1-1* and *EjSOC1-2* were abundantly expressed in the normal growth group and the control group at the end of June and early July (Figure 5A). More importantly, the SD-treated and GA_3_-treated loquat trees did not produce visible inflorescences in September compared to the trees in the normal growth group and the control group (Figure 5B). Furthermore, according to qPCR, the floral meristem identity genes *EjAP1-1* and *EjAP1-2* were hardly expressed (Figure 5A), as well as *EjLFY-1* and *EjLFY-2* were consistently expressed at a low level (Figure 5A). Based on the above results, we conclude that *EjSOC1-1* and *EjSOC1-2* are regulated by photoperiod and GA_3_ and that flower bud differentiation does not occur under SD conditions or after GA_3_ exposure. The results suggest that *EjSOC1*s can initiate flower bud differentiation by integrating photoperiod and gibberellin signaling.

### Subcellular Localization of EjSOC1s

**FIGURE 6:**
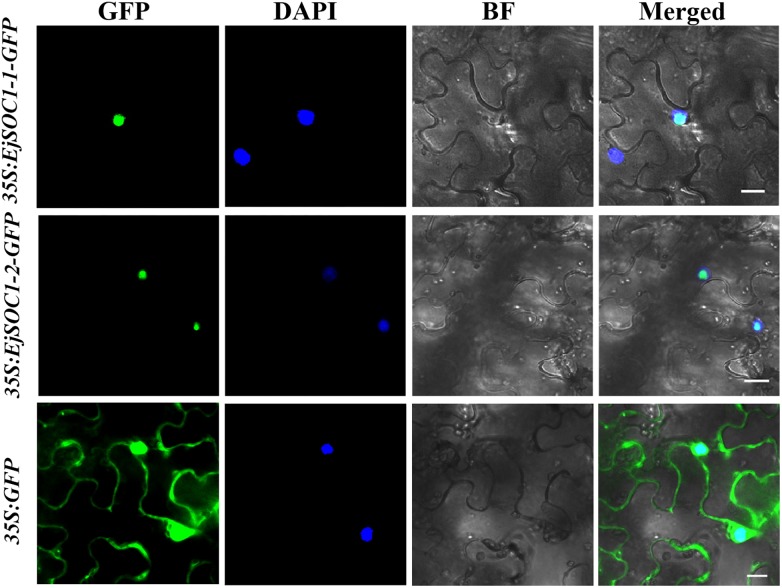
Subcellular localization of EjSOC1s. GFP, GFP fluorescence; 4,6-diamidino-2-phenylindole (DAPI) staining indicates nuclear localization; BF, bright-field; Merged, merged image of GFP and DAPI. Scale bars = 20 μm.

To examine the subcellular localization of EjSOC1-1 and EjSOC1-2, 35S-EjSOC1-1-GFP, and 35S-EjSOC1-2-GFP fusion proteins were generated and transiently expressed in leaf epidermal cells of *N. benthamiana*. Fluorescence from the 35S-GFP control was detected in both the cytoplasm and nucleus, whereas fluorescence from the 35S-EjSOC1-1-GFP and 35S-EjSOC1-2-GFP fusions was detected only in the nucleus (Figure 6). These results indicate that EjSOC1-1 and EjSOC1-2 were nuclear-localized proteins. These subcellular localization patterns were similar to AtSOC1 in *Arabidopsis* (Lee et al., 2008).

### Functional Analysis of EjSOC1s in *Arabidopsis*

**FIGURE 7:**
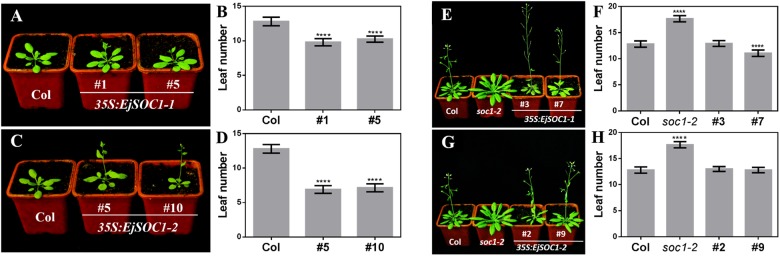
Overexpression of *EjSOC1*s in *Arabidopsis* accelerates flowering. **(A)** *35S:EjSOC1-1-HA* transgenic plants exhibit earlier flowering than wild-type Col-0 plants. **(B)** Rosette leaf number of Col-0 and *35S:EjSOC1-1-HA* transgenic plants. **(C)** *35S:EjSOC1-2-HA* transgenic plants exhibit earlier flowering than wild-type Col-0 plants. **(D)** Rosette leaf number of Col-0 and *35S:EjSOC1-1-HA* transgenic plants. **(E)** *35S:EjSOC1-1-HA*/+*soc1-2* transgenic plants exhibit earlier flowering than *soc1-2* mutant plants. **(F)** Rosette leaf number of Col-0, *soc1-2* mutant and *35S:EjSOC1-1-HA*/+*soc1-2* transgenic plants. **(G)** *35S:EjSOC1-2-HA*/+*soc1-2* transgenic plants exhibit earlier flowering than *soc1-2* mutant plants. **(H)** Rosette leaf number of Col-0, *soc1-2* mutant and *35S:EjSOC1-2-HA*/+*soc1-2* transgenic plants. Asterisks indicate significant differences between Col-0, *soc1-2* mutant and transgenic plants (*n* ≥ 20; error bars indicating SD from three biological replicates; ^∗∗∗∗^are significantly different from WT at *p* < 0.0001, by Student’s *t*-test).

To examine whether *EjSOC1-1* and *EjSOC1-2* encode functional homologs of *AtSOC1*, we generated *35S:EjSOC1-1-HA* and *35S:EjSOC1-2-HA* constructs and introduced them into *Arabidopsis* wild-type ecotype Col-0 and the late flowering mutant *soc1-2*. Although the wild-type plants flowered when 13 or 14 rosette leaves appeared, the *35S:EjSOC1-1-HA* and *35S:EjSOC1-2-HA* transgenic lines flowered with only 6–10 rosette leaves (Figure 7A–D). In addition, *soc1-2* mutant, which showed an obviously delayed phenotype compared to wild-type, flowered with 17 or 18 rosette leaves. However, in *35S:EjSOC1-1-HA*/+*soc1-2-* and *35S:EjSOC1-2-HA*/+*soc1-2*-overexpressing lines, flowering occurred at a comparable or even lower number of rosette leaves compared to wild-type (Figure 7E–H). We also detected expression of *EjSCO1*s in the transgenic plants and found high levels in the respective lines (10 and 20 days) (Supplementary Figures S5A–C). Compared to Col-0, both *AtAP1* and *AtLFY* were relatively highly expressed in the *35S:EjSOC1*s*-HA* transgenic lines (Supplementary Figures S5D,E), and the expression levels of *AtAP1* and *AtLFY* in the *35S:EjSOC1*s*-HA*/+*soc1-2* transgenic line and Col-0 were similar or even higher than the expression level in Col-0 (Supplementary Figures S5F,G). These results suggest that *EjSOC1-1* and *EjSOC1-2* both have a conserved role in accelerating flowering in *Arabidopsis* and that they may have a significant function in inducing flowering in loquat.

**FIGURE 8:**
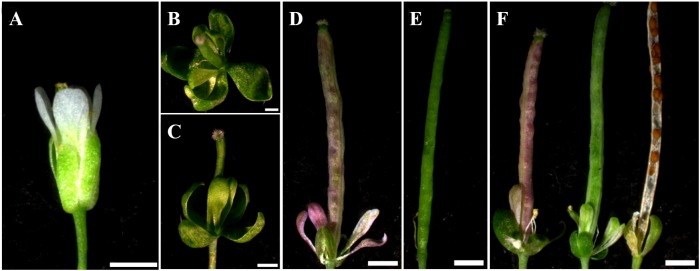
Phenotypes of flowers and siliques of Col-0 and *35S:EjSOC1-2-HA* transgenic plants. **(A)** Normal flower development of wild-type *Arabidopsis* Col-0. **(B–D)** Abnormal phenotypes of flowers and siliques of transgenic plants. **(E)** Normal phenotypes of wild-type *Arabidopsis* Col-0 siliques. **(D,F)** Abnormal phenotypes of transgenic plant siliques. Scale bars = 5 mm.

Interestingly, different from the Col-0 phenotypes (Figure 8A,E), a number of flower and silique phenotypes were observed in the *35S:EjSOC1-2-HA* transgenic lines. For example, some of the petals were green or lilac in color; hypogenetic stamens were also observed, and the calyx showed anomalous growth (Figure 8B–D) and was not shed after maturity (Figure 8F). Additionally, the surface of some siliques was lilac (Figure 8D,F).

## Discussion

The phenomenon of flowering in autumn and harvesting in spring is very unique in rosaceous plants. In spring, other fresh fruits are rarely sold, or the price of fruit is relatively high, and the range of choices for fruits is greatly reduced. Loquat is undoubtedly a relatively healthy and delicious choice in the fruit market at this time of year. In addition, loquat fruit is affected by storage and transportation. However, loquat fruits are easily injured and only remain fresh for 10 days at normal temperatures (Lin, 2007). Therefore, the price of loquat fruits is usually high, and their transport time to market is short. Overall, determining the flowering time and flowering mechanism of loquat will provide a means to successfully advance or delay it. If that were the case, loquat may be cultivated for a longer period of time and would be more stable in the fruit market for a longer duration. Notably, *Eriobotrya* species vary in flowering time, but each species of this genus can be hybridized, and fertile offspring can be obtained (Lin, 2017). This fact suggests that the flowering period of loquat is flexible. Research on the flowering mechanism of loquat can greatly benefit loquat growers and, more importantly, provide us with new insight into perennial fruit breeding.

*SOC1*, which can integrate the gibberellin pathway, photoperiod pathway and age pathway, is a key integration factor in flowering. In the age pathway, miRNA156 is highly expressed in juveniles and inhibits transcription of the SBP family transcription factor *SQUAMOSA PROMOTER BINDING PROTEIN LIKE (SPL)*. As plants mature, expression of miRNA156 decreases, the transcriptional level of *SPL*s increases, and SPL15 binds to *SOC1* in a GA-dependent manner, recruiting MED18 and RNAPII to induce expression of the downstream MADS-box flowering gene *FRUITFULL (FUL)*, which promotes flowering (Hyun et al., 2016). In the photoperiod pathway, FT activates expression of the flowering integration gene *SOC1* and the floral meristem gene *APETALA (AP1)* to initiate flower bud differentiation and flower development (Abe et al., 2005; Wigge et al., 2005). In the gibberellic acid (GA) pathway, the MADS-box transcription factor AGAMOUS-LIKE24 (AGL24) interacts with SOC1, resulting in direct transcriptional upregulation of both (Liu et al., 2008). Under SD conditions, *FT* expression is low, and AGL24 interacts with SOC1 to promote *Arabidopsis* flowering (Liu et al., 2008; Tao et al., 2012). In addition, nuclear factor Y (NF-Y) interacts with the photoperiod transcription factor CO and the GA pathway transcription factor DELLA, directly binding to a unique *cis*-element within the *SOC1* gene, regulates H3K27 methylation levels of *SOC1*, and affects flowering time (Hou et al., 2014). Unlike in *Arabidopsis*, studies on woody fruit trees have shown that GA_3_ inhibits floral bud induction (García-Pallas et al., 2001; Lenahan et al., 2006; Nakagawa et al., 2012; Goldberg-Moeller et al., 2013; Zhang S. et al., 2016).

In this study, the possibility that EjSOC1s are involved in loquat flower formation was identified, and we found that *EjSOC1* expression is regulated by photoperiod and GA_3_. More importantly, our results show that SD and GA_3_ treatment can inhibit flower differentiation in loquat. A detailed analysis of the gibberellin pathway and photoperiod pathway will help us to better understand the biological mechanism of flower bud differentiation in loquat and provide new insight into artificially delaying flowering in woody fruit trees.

There is increasing evidence that the initiation of flower buds is mainly regulated by *AP1* and *LFY*, of which *AP1* is mainly regulated by *FT* and *LFY* mainly by *SOC1* (Abe et al., 2005; Lee et al., 2008). Furthermore, chromatin immunoprecipitation analysis indicated that the SOC1 protein can directly bind to the CArG domain in the *LFY* promoter (Lee et al., 2008; Liu et al., 2008). In this study, expression of *EjAP1-1*, *EjAP1-2*, *EjLFY-1*, and *EjLFY-2* began to increase in late June and early July, and observation of paraffin-embedded sections showed that the leaf buds began to differentiate into flower buds from late June to early July. These results are consistent and show that *EjAP1-1*, *EjAP1-2*, *EjLFY-1*, or *EjLFY-2* may be used as markers for identifying flower bud differentiation in loquat. In addition, the expression trends of *EjSOC1*s (Figure 4D,E) and *EjLFY*s (Figure 1C) in the SAM were similar, with *EjSOC1-1* and *EjSOC1-2* beginning to be highly expressed only 10 days earlier than *EjLFY*s. In addition, heterologous overexpression of *EjSOC1*s in *Arabidopsis* significantly upregulated expression of *AtLFY*.

Furthermore, it is worth noting that, *EjSOC1-1* and *EjSOC1-2* were differentially expressed at different developmental stages in leaves, *EjSOC1-1* transcription level increased obviously as the young leaves getting mature, and decreased in the late stage of leaf development, this implied *EjSOC1-1* might attend the regulation of leaf development, however, the expression of *EjSOC1-2* did not show distinct variation. It was reported that aging transcription factor *AtSPLs* can up-regulate *AtSOC1*, and therefore promote flowering in *Arabidopsis* (Wang et al., 2009). In loquat flower, *EjSOC1-1* and *EjSOC1-2* were mainly expressed in receptacle and pistil, respectively. We speculated that *EjSOC1*s have a positive effect on the development of floral organs. In the overexpressed transgenic *Arabidopsis*, phenotypes including color changed petal and silique suggested that EjSOC1-2 might interrupt normal flower development through the abnormal regulation, in addition, the changed color of petals showed possible function of EjSOC1-2 on secondary metabolism, which is worth to investigate in the future. These results provide a theoretical basis for further exploration of the function and mechanism of *EjSOC1*s in loquat growth and development.

In recent research, *EjFT1* has been shown to possibly have to do with bud sprouting and leaf development, whereas *EjFT2* has been shown to possibly be involved in flower bud induction (Reig et al., 2017). In this study, *EjSOC1-1* and *EjSOC1-2* showed different expression trends in leaves with different levels of maturity in the same period (Figure 4B). Therefore, it is speculated that *EjSOC1-1* is involved in the processes of flower development and leaf growth. In addition, the expression levels of *EjSOC1-1* and *EjSOC1-2* differed in various flower tissues (Figure 4F). Clearly, *EjSOC1-1* and *EjSOC1-2* have some different functions. In the model plant *Arabidopsis*, SOC1 integrates the photoperiod pathway through FT, which is transported to the SAM and interacts with FD to upregulate SOC1 (Lee and Lee, 2010). Similarly, in loquat (*Eriobotrya deflexa* Nakai f. *koshunensis*), EdFT can interact with both EdFD1 and EdFD2 (Zhang L. et al., 2016). These interesting and meaningful findings provide a basis for further studies on the growth and development of loquat and a reference for such studies in other species.

## Author Contributions

YyJ, SL, and YG designed the research. YyJ mainly performed the research. JP, YZ, WS, LZ, and YiJ finished some parts of the experiments. YyJ wrote the manuscript. SL and YG revised and approved the manuscript.

## Conflict of Interest Statement

The authors declare that the research was conducted in the absence of any commercial or financial relationships that could be construed as a potential conflict of interest.

## References

[B1] Abe M. Kobayashi Y. Yamamoto S. Daimon Y. Yamaguchi A. Ikeda Y. (2005). FD, a bZIP protein mediating signals from the floral pathway integrator FT at the shoot apex. *Science* 309 1052–1056. 10.1126/science.1115983 16099979

[B2] Amasino R. (2010). Seasonal and developmental timing of flowering. *Plant J.* 61 1001–1013. 10.1111/j.1365-313X.2010.04148.x 20409274

[B3] Chen F. Zhang X. Liu X. Zhang L. (2017). Evolutionary analysis of MIKC(c)-Type MADS-box genes in gymnosperms and angiosperms. *Front. Plant Sci.* 8:895. 10.3389/fpls.2017.00895 28611810 PMC5447709

[B4] Esumi T. Tao R. Yonemori K. (2005). Isolation of leafy and terminal flower 1 homologues from six fruit tree species in the subfamily Maloideae of the Rosaceae. *Sex. Plant Reprod.* 17 277–287. 10.1007/s00497-004-0239-3

[B5] Ferrario S. Busscher J. Franken J. Gerats T. Vandenbussche M. Angenent G. C. (2004). Ectopic expression of the petunia MADS box gene UNSHAVEN accelerates flowering and confers leaf-like characteristics to floral organs in a dominant-negative manner. *Plant Cell* 16 1490–1505. 10.1105/tpc.019679 15155884 PMC490041

[B6] García-Pallas I. Val J. Blanco A. (2001). The inhibition of flower bud differentiation in ‘Crimson Gold’ nectarine with GA3 as an alternative to hand thinning. *Sci. Horticult.* 90 265–278. 10.1016/S0304-4238(01)00229-1

[B7] Goldberg-Moeller R. Shalom L. Shlizerman L. Samuels S. Zur N. Ophir R. (2013). Effects of gibberellin treatment during flowering induction period on global gene expression and the transcription of flowering-control genes in *Citrus buds*. *Plant Sci.* 198 46–57. 10.1016/j.plantsci.2012.09.012 23199686

[B8] Gu C. Spongberg S. A. (2003). “ERIOBOTRYA lindley,” in *Flora of China*, eds Zhengyi W. Raven P. H. Hong D. Y. (Jefferson, MO: Missouri Botanical Garden Press).

[B9] Hong S. M. Bahn S. C. Lyu A. Jung H. S. Ahn J. H. (2010). Identification and testing of superior reference genes for a starting pool of transcript normalization in Arabidopsis. *Plant Cell Physiol.* 51 1694–1706. 10.1093/pcp/pcq128 20798276

[B10] Hou X. Zhou J. Liu C. Liu L. Shen L. Yu H. (2014). Nuclear factor Y-mediated H3K27me3 demethylation of the SOC1 locus orchestrates flowering responses of *Arabidopsis*. *Nat. Commun.* 5:4601. 10.1038/ncomms5601 25105952

[B11] Hyun Y. Richter R. Vincent C. Martinez-Gallegos R. Porri A. Coupland G. (2016). Multi-layered regulation of SPL15 and cooperation with SOC1 integrate endogenous flowering pathways at the *Arabidopsis* shoot meristem. *Dev. Cell* 37 254–266. 10.1016/j.devcel.2016.04.001 27134142

[B12] Kurokura T. Mimida N. Battey N. H. Hytonen T. (2013). The regulation of seasonal flowering in the Rosaceae. *J. Exp. Bot.* 64 4131–4141. 10.1093/jxb/ert233 23929655

[B13] Lee J. Lee I. (2010). Regulation and function of SOC1, a flowering pathway integrator. *J. Exp. Bot.* 61 2247–2254. 10.1093/jxb/erq098 20413527

[B14] Lee J. Oh M. Park H. Lee I. (2008). SOC1 translocated to the nucleus by interaction with AGL24 directly regulates leafy. *Plant J.* 55 832–843. 10.1111/j.1365-313X.2008.03552.x 18466303

[B15] Lee L. Y. Hou X. Fang L. Fan S. Kumar P. P. Yu H. (2012). *STUNTED* mediates the control of cell proliferation by GA in *Arabidopsis*. *Development* 139 1568–1576. 10.1242/dev.079426 22492352

[B16] Lenahan O. M. Whiting M. D. Elfving D. C. (2006). Gibberellic acid inhibits floral bud induction and improves ‘Bing’ sweet cherry fruit quality. *Hortscience* 41 654–659.

[B17] Lin S. Q. (2007). World loquat production and research with special reference to China. *Acta Hortic.* 750 37–44. 10.17660/ActaHortic.2007.750.2

[B18] Lin S. Q. (2017). A review on research of the wild species in genus eriobotrya germplasm and their innovative utilization. *Acta Hortic. Sin.* 44 1704–1716. 10.16420/j.issn.0513-353x

[B19] Liu C. Chen H. Er H. L. Soo H. M. Kumar P. P. Han J. H. (2008). Direct interaction of *AGL24* and *SOC1* integrates flowering signals in *Arabidopsis*. *Development* 135 1481–1491. 10.1242/dev.020255 18339670

[B20] Liu C. Zhu C. Zeng H. M. (2016). Key KdSOC1 gene expression profiles during plantlet morphogenesis under hormone, photoperiod, and drought treatments. *Genet Mol. Res.* 15 gmr7579. 10.4238/gmr.15017579 26909971

[B21] Liu Y. Song H. Liu Z. Hu G. Lin S. (2013). Molecular characterization of loquat EjAP1 gene in relation to flowering. *Plant Growth Regul.* 70 287–296. 10.1007/s10725-013-9800-0

[B22] Liu Y. Zhao Q. Meng N. Song H. Li C. Hu G. (2017). Over-expression of EjLFY-1 leads to an early flowering habit in strawberry (Fragaria x ananassa) and Its asexual progeny. *Front. Plant Sci.* 8:496. 10.3389/fpls.2017.00496 28443106 PMC5385365

[B23] Lohmann J. U. Hong R. L. Hobe M. Busch M. A. Parcy F. Simon R. (2001). A molecular link between stem cell regulation and floral patterning in *Arabidopsis*. *Cell* 105 793–803. 10.1016/s0092-8674(01)00384-1 11440721

[B24] Moon J. Lee H. Kim M. Lee I. (2005). Analysis of flowering pathway integrators in *Arabidopsis*. *Plant Cell Physiol.* 46 292–299. 10.1093/pcp/pci024 15695467

[B25] Mouhu K. Kurokura T. Koskela E. A. Albert V. A. Elomaa P. Hytonen T. (2013). The *Fragaria vesca* homolog of suppressor of overexpression of constans1 represses flowering and promotes vegetative growth. *Plant Cell* 25 3296–3310. 10.1105/tpc.113.115055 24038650 PMC3809533

[B26] Nakagawa M. Honsho C. Kanzaki S. Shimizu K. Utsunomiya N. (2012). Isolation and expression analysis of FLOWERING LOCUS T-like and gibberellin metabolism genes in biennial-bearing mango trees. *Sci. Hortic.* 139 108–117. 10.1016/j.scienta.2012.03.005

[B27] Parcy F. (2005). Flowering: a time for integration. *Int. J. Dev. Biol.* 49 585–593. 10.1387/ijdb.041930fp 16096967

[B28] Parenicova L. de Folter S. Kieffer M. Horner D. S. Favalli C. Busscher J. (2003). Molecular and phylogenetic analyses of the complete MADS-box transcription factor family in Arabidopsis: new openings to the MADS world. *Plant Cell* 15 1538–1551. 10.1105/tpc.011544 12837945 PMC165399

[B29] Reig C. Gil-Munoz F. Vera-Sirera F. Garcia-Lorca A. Martinez-Fuentes A. Mesejo C. (2017). Bud sprouting and floral induction and expression of FT in loquat [Eriobotrya japonica (Thunb.) Lindl.]. *Planta* 246 915–925. 10.1007/s00425-017-2740-6 28710586

[B30] Ruokolainen S. Ng Y. P. Albert V. A. Elomaa P. Teeri T. H. (2011). Over-expression of the *Gerbera hybrida* At-SOC1-like1 gene Gh-SOC1 leads to floral organ identity deterioration. *Ann. Bot.* 107 1491–1499. 10.1093/aob/mcr112 21572092 PMC3108810

[B31] Shan L. L. Li X. Wang P. Cai C. Zhang B. Sun C. D. (2008). Characterization of cDNAs associated with lignification and their expression profiles in loquat fruit with different lignin accumulation. *Planta* 227 1243–1254. 10.1007/s00425-008-0696-2 18273642

[B32] Smaczniak C. Immink R. G. Angenent G. C. Kaufmann K. (2012). Developmental and evolutionary diversity of plant MADS-domain factors: insights from recent studies. *Development* 139 3081–3098. 10.1242/dev.074674 22872082

[B33] Sparkes I. A. Runions J. Kearns A. Hawes C. (2006). Rapid, transient expression of fluorescent fusion proteins in tobacco plants and generation of stably transformed plants. *Nat. Protoc.* 1 2019–2025. 10.1038/nprot.2006.286 17487191

[B34] Sri T. Mayee P. Singh A. (2015). Sequence and expression variation in suppressor of overexpression of constans 1 (SOC1): homeolog evolution in Indian Brassicas. *Dev. Genes Evol.* 225 287–303. 10.1007/s00427-015-0513-4 26276216

[B35] Srikanth A. Schmid M. (2011). Regulation of flowering time: all roads lead to Rome. *Cell Mol. Life Sci.* 68 2013–2037. 10.1007/s00018-011-0673-y 21611891 PMC11115107

[B36] Tadege M. Sheldon C. C. Helliwell C. A. Upadhyaya N. M. Dennis E. S. Peacock W. J. (2003). Reciprocal control of flowering time by OsSOC1 in transgenic *Arabidopsis* and by FLC in transgenic rice. *Plant Biotechnol. J.* 1 361–369. 10.1046/j.1467-7652.2003.00034.x 17166135

[B37] Tan F. C. Swain S. M. (2007). Functional characterization of AP3, SOC1 and WUS homologues from citrus (Citrus sinensis). *Physiol. Plant* 131 481–495. 10.1111/j.1399-3054.2007.00971.x 18251886

[B38] Tao Z. Shen L. S. Liu C. Liu L. Yan Y. Y. Yu H. (2012). Genome-wide identification of SOC1 and SVP targets during the floral transition in Arabidopsis. *Plant J.* 70 549–561. 10.1111/j.1365-313X.2012.04919.x 22268548

[B39] Teotia S. Tang G. (2015). To bloom or not to bloom: role of microRNAs in plant flowering. *Mol. Plant* 8 359–377. 10.1016/j.molp.2014.12.018 25737467

[B40] Theissen G. Kim J. T. Saedler H. (1996). Classification and phylogeny of the MADS-box multigene family suggest defined roles of MADS-box gene subfamilies in the morphological evolution of eukaryotes. *J. Mol. Evol.* 43 484–516. 8875863 10.1007/BF02337521

[B41] Vandenbussche M. Theissen G. Van de Peer Y. Gerats T. (2003). Structural diversification and neo-functionalization during floral MADS-box gene evolution by C-terminal frameshift mutations. *Nucleic Acids Res.* 31 4401–4409. 10.1093/nar/gkg642 12888499 PMC169922

[B42] Voogd C. Wang T. Varkonyi-Gasic E. (2015). Functional and expression analyses of kiwifruit SOC1-like genes suggest that they may not have a role in the transition to flowering but may affect the duration of dormancy. *J. Exp. Bot.* 66 4699–4710. 10.1093/jxb/erv234 25979999 PMC4507769

[B43] Wang J. W. Czech B. Weigel D. (2009). miR156-regulated SPL transcription factors define an endogenous flowering pathway in *Arabidopsis thaliana*. *Cell* 138 738–749. 10.1016/j.cell.2009.06.014 19703399

[B44] Wei J. Liu D. Liu G. Tang J. Chen Y. (2016). Molecular cloning, characterization, and expression of MiSOC1: a homolog of the flowering gene suppressor of overexpression of constans1 from mango (*Mangifera indica* L). *Front. Plant Sci.* 7:1758. 10.3389/fpls.2016.01758 27965680 PMC5126060

[B45] Wigge P. A. Kim M. C. Jaeger K. E. Busch W. Schmid M. Lohmann J. U. (2005). Integration of spatial and temporal information during floral induction in *Arabidopsis*. *Science* 309 1056–1059. 10.1126/science.1114358 16099980

[B46] Zhang L. Yu H. Lin S. Gao Y. (2016). Molecular characterization of FT and FD homologs from eriobotrya deflexa nakai forma koshunensis. *Front. Plant Sci.* 7:8. 10.3389/fpls.2016.00008 26834775 PMC4722113

[B47] Zhang S. Zhang D. Fan S. Du L. Shen Y. Xing L. (2016). Effect of exogenous GA3 and its inhibitor paclobutrazol on floral formation, endogenous hormones, and flowering-associated genes in ‘Fuji’ apple (Malus domestica Borkh.). *Plant Physiol. Biochem.* 107 178–186. 10.1016/j.plaphy.2016.06.005 27295342

[B48] Zhang X. Henriques R. Lin S. S. Niu Q. W. Chua N. H. (2006). Agrobacterium-mediated transformation of *Arabidopsis thaliana* using the floral dip method. *Nat. Protoc.* 1 641–646. 10.1038/nprot.2006.97 17406292

[B49] Zhao S. Luo Y. Zhang Z. Xu M. Wang W. Zhao Y. (2014). ZmSOC1, a MADS-box transcription factor from *Zea mays*, promotes flowering in *Arabidopsis*. *Int. J. Mol. Sci.* 15 19987–20003. 10.3390/ijms151119987 25372944 PMC4264151

[B50] Zhong X. Dai X. Xv J. Wu H. Liu B. Li H. (2012). Cloning and expression analysis of GmGAL1, SOC1 homolog gene in soybean. *Mol. Biol. Rep.* 39 6967–6974. 10.1007/s11033-012-1524-0 22350155

