# Supplementary material for: The Role of EjSOC1s in Flower Initiation in Eriobotrya japonica
Source: Front Plant Sci. 2019 Mar 4;10:253. doi: 10.3389/fpls.2019.00253 (PMC6409497; doi:10.3389/fpls.2019.00253)
Supplement: Supplementary file 1 [file Data_Sheet_1.pdf]

Supplementary Material

The role of *EjSOC1*s in flower initiation in *Eriobotrya japonica*

Yuanyuan Jiang[1], Jiangrong Peng[1], Yunmei Zhu[1], Wenbing SU[1], Ling Zhang[1], Yi Jing[2], Shunquan Lin[1]* and Yongshun Gao[1]*

**** Correspondence:***

Corresponding Author: loquat@scau.edu.cn; yongshungao@163.com

Table S1. Primers used for gene cloning.

Primer name	Sequence (5'-3')
EjSOC1-1-FP	ATGGTGAGAGGAAAAACTC
EjSOC1-1-RP	CTAGCGCCTCGCCCTACTTTC
EjSOC1-2-FP	ATGGTGAGAGGAAAAACTCAG
EjSOC1-2-RP	CTAGCGCCTAGCTCTACTTTC

Table S2. Primers used for the analysis of gene expression by qPCR.

Primer name	Sequence (5'-3')
β-actin-FP	GGATTTGCTGGTGATGATGC
β-actin-RP	CCGTGCTCAATGGGATACTT
EjSOC1-1-FP	GCGTTATCAGAAGCATGCGAAAGA
EjSOC1-1-RP	GCTCGGACGTTGTAGACGCTC
EjSOC1-2-FP	GAGCGATATCAGAATCACGC
EjSOC1-2-RP	GGATCGGACGTTGTTCACGC
EjSOC1-1-RT-FP	ATGGTGAGAGGAAAAACTC
EjSOC1-1- RT-RP	GCGCCTCGCCCTACTTTC
EjSOC1-2- RT-FP	ATGGTGAGAGGAAAAACTCAG
EjSOC1-2- RT-RP	GCGCCTAGCTCTACTTTC
EjAP1-1-FP	TGATGCTCAAGTTGCTGTGG
EjAP1-1-RP	TGCATGAATCTGTGGCGTAC

EjAP1-2-FP	CCTTCCATGTCTTAACATT
EjAP1-2-RP	AAATGGTTCCAGAGTAAGGT
EjLFY-1-FP	GGGTCATGATCATAACGAG
EjLFY-1-RP	TCTGTCTCTCGCCTAGCAACC
EjLFY-2-FP	GGATGACAACGACATGGACG
EjLFY-2-RP	GGTGCTCCCTTTGTCTCTCT
AtPP2AA3-FP	GCGGTTGTGGAGAACATGATACG
AtPP2AA3-RP	GAACCAAACACAATTCGTTGCTG
AtAP1-FP	CATGGGTGGTCTGTATCAAGAAGAT
AtAP1-RP	CATGCGGCGAAGCAGCCAAGGTT
AtLFY-FP	ACGCCGTCATTTGCTACTCT
AtLFY-RP	CTTTCTCCGTCTCTGCTGCT

Table S3. Primers used for vector construction.

Vector Name	Primer name	Sequence (5'-3')
35S:EjSOC1-1-HA/	*EjSOC1-1*-HindIII	GTCGACGGTATCGATAAGCTTATGGTGAGAGGAAAAAC
35S:EjSOC1-1-GFP	*EjSOC1-1*-EcoRI	TCCCCCGGGCTGCAGGAATTCGCGCCTCGCCCTACTT
35S:EjSOC1-2-HA/	*EjSOC1-2*-HindIII	GTCGACGGTATCGATAAGCTTATGGTGAGAGGAAAAAC
35S:EjSOC1-2-GFP	*EjSOC1-2*-EcoRI	TCCCCCGGGCTGCAGGAATTCGCGCCTAGCTCTACTT

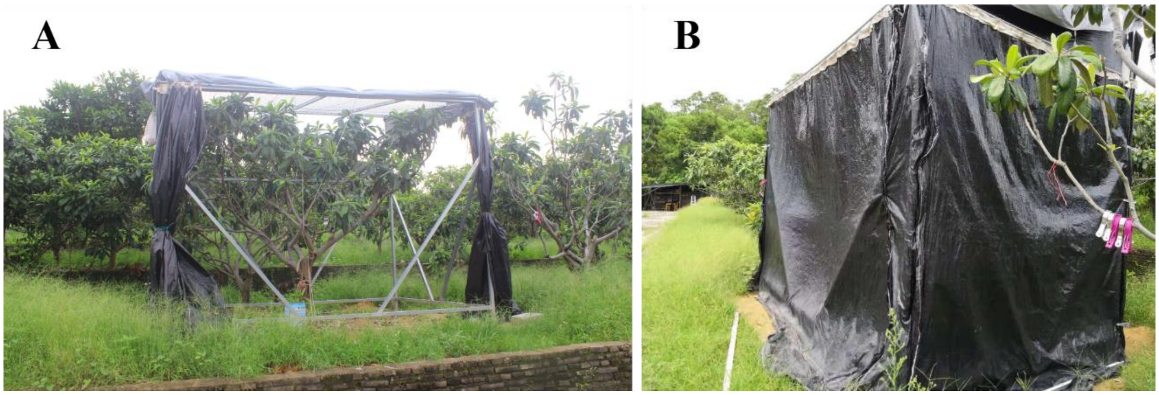

Figure S1. The picture of awning.

EjSOC1-1

```
1      ATGGTGAGAGGAAAAACTCAGATGAGGCGCATAGAGAACACGACGAGTCGTCAGGTGACATTCTCAAAGAGGAGAAGTGGTCTGCTAAAG
1       M  V  R  G  K  T  Q  M  R  R  I  E  N  T  T  S  R  Q  V  T  F  S  K  R  R  S  G  L  L  K

91     AAGGCCTTTGAGCTCTCAGTTCTTTGCGAAGCTGAGGTTTCCCTGATCATCTTCTCTCCCAGAGGAAAACTCTACGAATTTGCAAGTTCC
31      K  A  F  E  L  S  V  L  C  E  A  E  V  S  L  I  I  F  S  P  R  G  K  L  Y  E  F  A  S  S

181    AGCATGCAGGGAACCATAGAGCGTTATCAGAAGCATGCGAAAGACAATCAAACCAACAACAAATCCGGTTCCAGTGAACAAAATATGCAG
61      S  M  Q  G  T  I  E  R  Y  Q  K  H  A  K  D  N  Q  T  N  N  K  S  G  S  S  E  Q  N  M  Q

271    CATCTGAAGCAAGAAGCAACTAGCATGATGAAGCAGATAGAGCTTCTTGAAGTATCAAAACGGAAACTCTTGGGAGAGGGTCTGGGATCA
91      H  L  K  Q  E  A  T  S  M  M  K  Q  I  E  L  L  E  V  S  K  R  K  L  L  G  E  G  L  G  S

361    TGCACTCTTGCAGAATTACAAGAAATAGAGCACCAGTTGGAGAAGAGCGTCTACAACGTCCGAGCCCGAAAGAGTCAGGTTTTCAAGGAA
121     C  T  L  A  E  L  Q  E  I  E  H  Q  L  E  K  S  V  Y  N  V  R  A  R  K  S  Q  V  F  K  E

451    CAGATTGAGCAACTGAGAGAAAAGGAAAAACTCCTCACAGCTGAAAATACAAGACTGGTTGAGAAGTATGGTAGTTTCAAGAAAACATTG
151     Q  I  E  Q  L  R  E  K  E  K  L  L  T  A  E  N  T  R  L  V  E  K  Y  G  S  F  K  K  T  L

541    GACGAGCGAAGAGAAAAGACACCCTACAATGAAAGTAGTACAAGCTCGGATGTTGAGACTGAATTGTTCATTGGACTGCCGGAAAGTAGG
181     D  E  R  R  E  K  T  P  Y  N  E  S  S  T  S  S  D  V  E  T  E  L  F  I  G  L  P  E  S  R

631    GCGAGGCGCTAG
211     A  R  R  *
```

EjSOC1-2

```
1      ATGGTGAGAGGAAAAACTCAGATGAGGCGCATAGAGAACGCGACGAGCCGTCAGGTGACATTCTCAAAGAGGAGAAGTGGTCTGCTAAAG
1       M  V  R  G  K  T  Q  M  R  R  I  E  N  A  T  S  R  Q  V  T  F  S  K  R  R  S  G  L  L  K

91     AAGGCCTTTGAGCTCTCAGTTCTTTGCGATGCTGAGGTTTCCCTCATCATCTTCTCTCCCAGAGGAAAACTCTTCGAATTTGCAAGTTCC
31      K  A  F  E  L  S  V  L  C  D  A  E  V  S  L  I  I  F  S  P  R  G  K  L  F  E  F  A  S  S

181    AGCATGCAGGGAACCATAGAGCGATATCAGAATCACGCAAAAGGCAATCAAACCAGCAACAAATCCAGTTCCAGTGAACAAAATATGCAG
61      S  M  Q  G  T  I  E  R  Y  Q  N  H  A  K  G  N  Q  T  S  N  K  S  S  S  S  E  Q  N  M  Q

271    CATCTGAAGCAAAAAGCAACTAGCATGATGAAACAGTTAGAGCTTCTTGAAGTATCAAAACGGAAACTCTTGGGAGAGGGTCTAGGATCA
91      H  L  K  Q  K  A  T  S  M  M  K  Q  L  E  L  L  E  V  S  K  R  K  L  L  G  E  G  L  G  S

361    TGCACTCTTGCAGAACTACAAGAAATAGAGCACCAGTTGGAGAAGAGCGTGAACAACGTCCGATCCCGAAAGAGTCAGGTTTTCAAGGAA
121     C  T  L  A  E  L  Q  E  I  E  H  Q  L  E  K  S  V  N  N  V  R  S  R  K  S  Q  V  F  K  E

451    CAGATTGAGCAACTGAGAGAAAAGGAGAAACTCCTCAAAGCTGAAACTGCACGACTGGTTGAGAAGTGTGGTAGTTTCCAGCCAAGGAAA
151     Q  I  E  Q  L  R  E  K  E  K  L  L  K  A  E  T  A  R  L  V  E  K  C  G  S  F  Q  P  R  K

541    ACATTGGATGAGCGAAGAGAAAACACAACCTACACTGATAGTAGTACAAGCTCAGATGTTGAGACTGAATTGTTCATTGGACCGCCAGAA
181     T  L  D  E  R  R  E  N  T  T  Y  T  D  S  S  T  S  S  D  V  E  T  E  L  F  I  G  P  P  E

631    AGTAGAGCTAGGCGCTAG
211     S  R  A  R  R  *
```

Figure S2. The nucleotide and deduced protein sequences of EjSOC1-1 and EjSOC1-2.

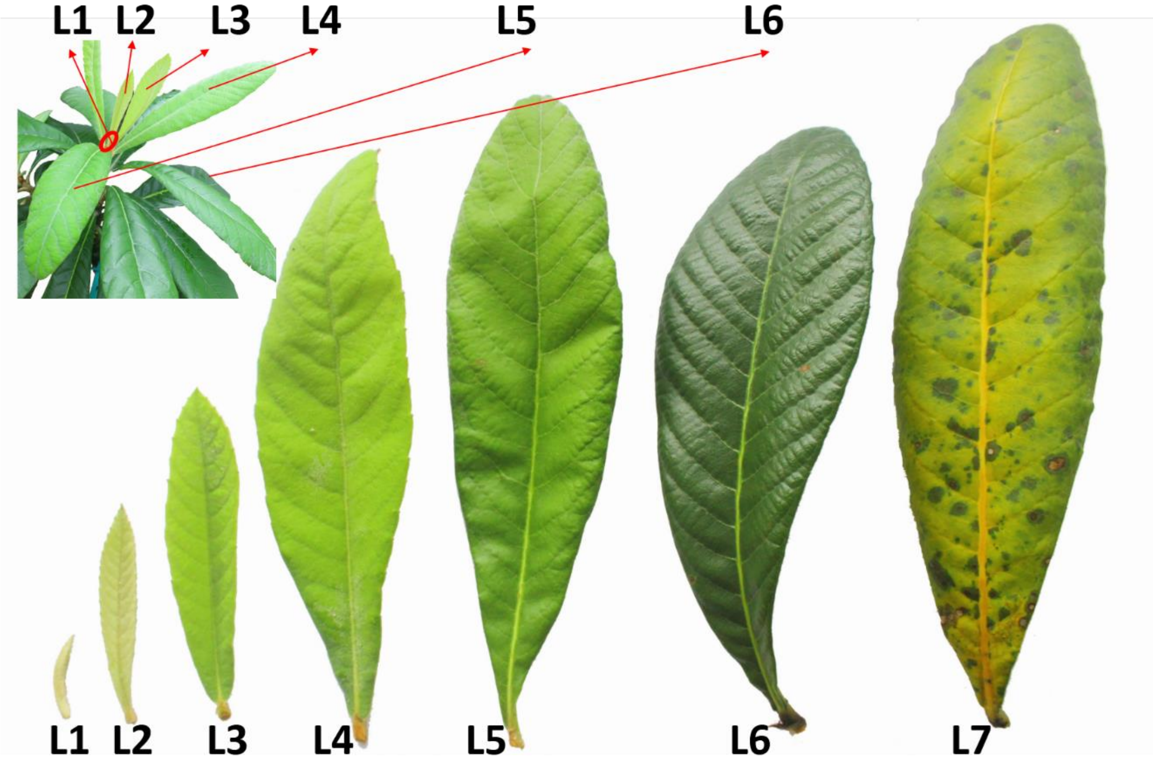

Figure S3. Leaves of different maturities in the same period. L1, L2, L3, L4, L5, L6 and L7 were taken from leaves of different maturities on June 9th, ‘L6’ indicated in maturity stage of leaves.

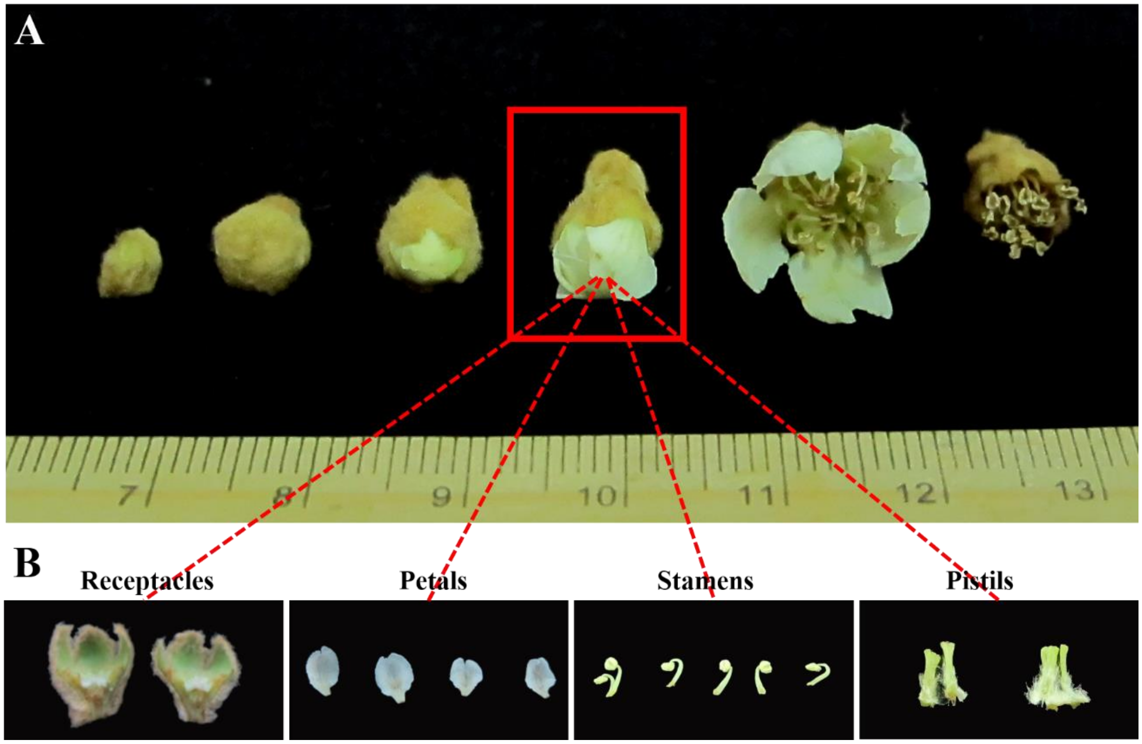

Figure S4. Different flower parts. (A) Different maturities flowers. The flower selected in the red frame is the experimental material. **(B)** Different flower parts.

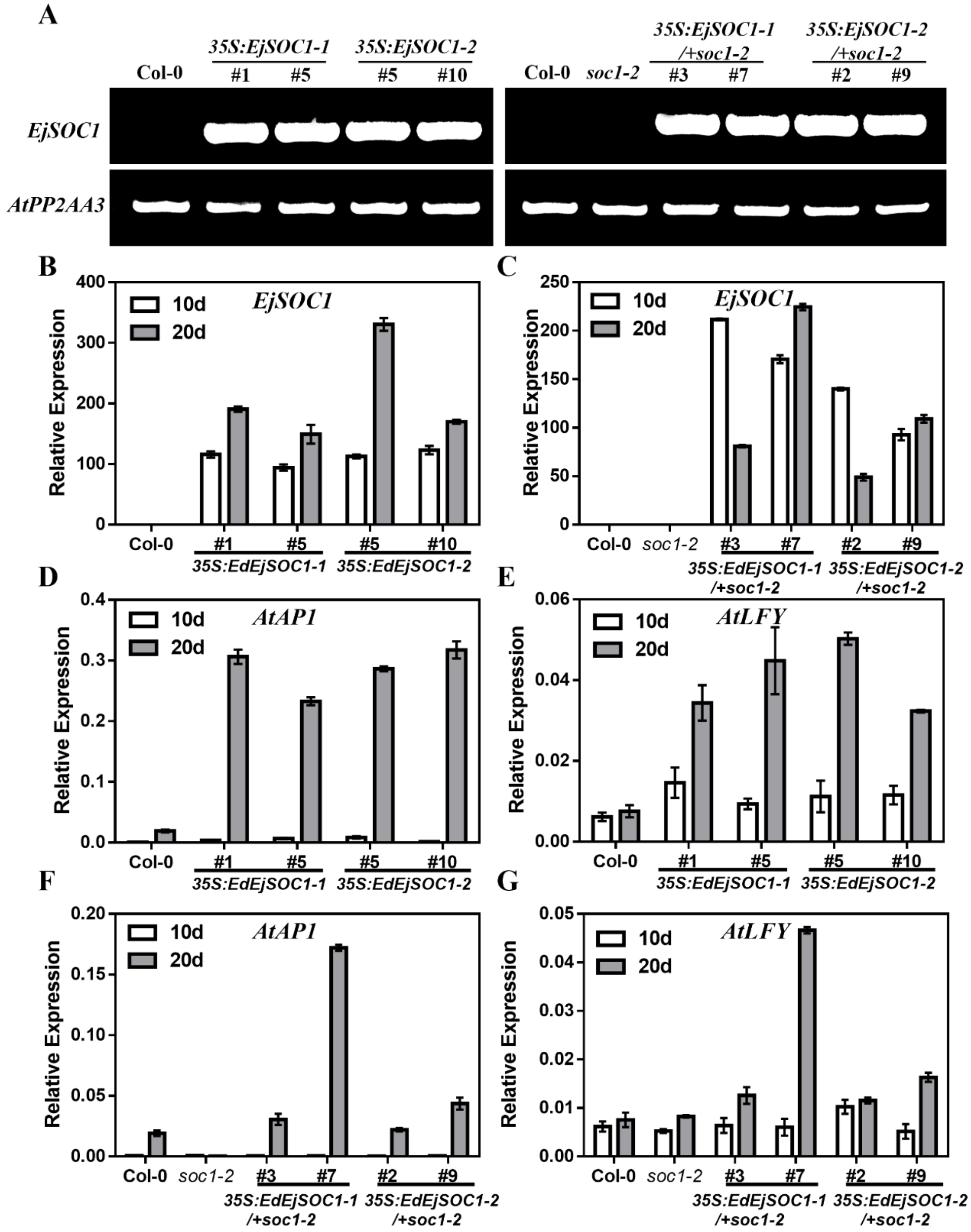

Figure S5. Relative expression levels of *EjSCO1*s, *AtAP1* and *AtLFY* in the transgenic plants. (A) Showed the RT-PCR results of *EjSOC1s* in ten-day-old transgenic plants. **(B)** Expression of *EjSOC1s* in ten-day-old and twenty-day-old Col and *35S:EjSOC1*-HA transgenic plants. **(C)** Expression of *EjSOC1s* in ten-day-old and twenty-day-old Col, *soc1-2* mutant and *35S:EjSOC1s-HA/+ soc1-2* transgenic plants. **(D)** Expression of *AtAP1* in ten-day-old and twenty-day-old Col and *35S:EjSOC1s*-HA transgenic plants. **(E)**

Expression of *AtLFY* in ten-day-old and twenty-day-old Col and *35S:EjSOC1s*-HA transgenic plants. **(F)** Expression of *AtAP1* in ten-day-old and twenty-day-old Col, *soc1-2* mutant and *35S:EjSOC1s-HA/+ soc1-2* transgenic plants. **(G)** Expression of *AtLFY* in ten-day-old and twenty-day-old Col, *soc1-2* mutant and *35S:EjSOC1s-HA/+ soc1-2* transgenic plants. Error bars indicating SD from three biological replicates.
